# Tuning protein synthesis for cancer therapy

**DOI:** 10.1080/23723556.2021.1884034

**Published:** 2021-02-22

**Authors:** John R. P. Knight, Owen J. Sansom

**Affiliations:** a CRUK Beatson Institute, University of Glasgow, Glasgow, UK; b Institute of Cancer Sciences, University of Glasgow, UK

**Keywords:** Protein synthesis, colorectal cancer, MYC, *KRAS* mutation, eIF4E

## Abstract

~50% of colorectal cancers have an activating mutation in *KRAS* (encoding the KRAS proto-oncogene) and remain difficult to target in the clinic. We have recently shown that activation of KRAS protein alters the regulation of mRNA translation, increasing total protein synthesis, and maintaining elevated c-MYC (MYC proto-oncogene) expression. Targeting these pathways downstream of KRAS reveals a striking dependency that has potential for clinical translation.

Mutations that result in the constitutive activation of KRAS signaling (KRAS proto-oncogene signaling) are present in nearly half of all colorectal cancers (CRCs), with these patients responding poorly to current treatments. The design of targeted therapies is reliant upon understanding how mutant KRAS alters tumor cells. We recently used genetically engineered mouse models and *KRAS*-mutant patient-derived organoids to investigate how KRAS activation alters the fundamental cellular process of protein synthesis.^1^ We found that activation of KRAS nearly doubled the rate of protein synthesis in organoids deficient for the tumor suppressor APC (APC regulator of WNT signaling pathway) (Figure 1). Furthermore, *Kras* mutation completely removed the efficacy of rapamycin as an inhibitor of proliferation in *Apc*-deficient cells, both in organoids and multiple *in vivo* models. This is in stark contrast to our previous work where targeting of mTORC1 (mammalian/mechanistic target of rapamycin complex 1) with rapamycin greatly suppressed intestinal adenoma proliferation, ^2^ and is in agreement with the drug resistance found in *KRAS*-mutant tumors in the clinic. An independent study in organoids of the same genotype as our mouse models found that *Kras* mutation alone has no effect on protein synthesis rates, but in conjunction with *Apc*-deficiency does lead to an increase.^3^ Interestingly, this same paper found further increases in protein synthesis upon suppression of the commonly mutated genes, *Smad4* (SMAD family member 4) and *Trp53* (transformation-related protein 53). This highlights a direct link between tumor mutations and protein synthesis that, when fully understood, could provide methods for personalized therapeutic intervention.

**Figure 1.**
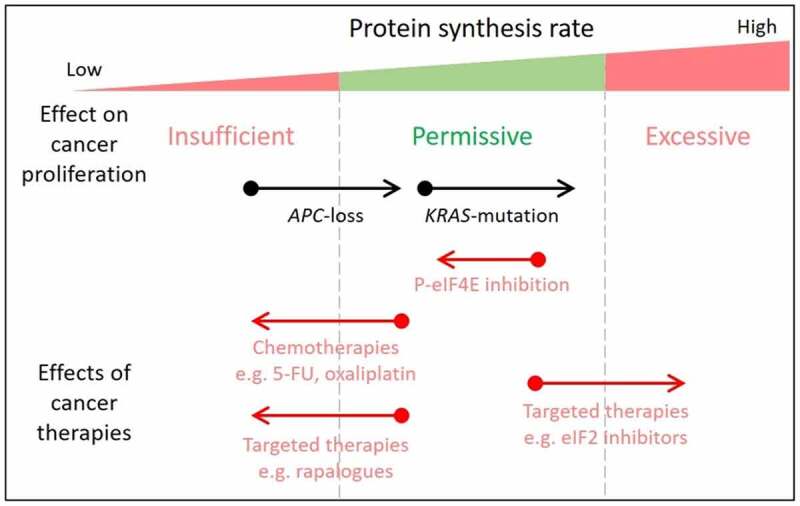
The permissive window for protein synthesis in cancer

Surprisingly, we found that rapamycin remains a potent inhibitor of protein synthesis in *Apc*-deficient, *Kras*-mutant mouse models, despite not affecting proliferation. From this, we draw two conclusions. First, that a proportion of translation occurring in *Kras*-mutant cells is not required for proliferation and, secondly, there is not a linear relationship between protein synthesis and proliferation rates. In agreement with this, targeting signaling to increase the rate of protein synthesis in CRC models via eIF2 (eukaryotic translation initiation factor 2) is effective at reducing proliferation and inducing apoptosis.^4^ In this instance increasing protein synthesis suppressed proliferation, highlighting the presence of a protein synthesis window that is permissive to cancer-associated hyper-proliferation (Figure 1). Clinical trials using compounds with this molecular mechanism have begun, with a view to using these in neurodegenerative diseases (NCT04268784). Pre-clinical studies show that these compounds may also be effective in cancer.

Our work found that the expression of mutant *Kras* in tumor models of the mouse intestinal epithelium increased the phosphorylation of eIF4E (eukaryotic translation initiation factor 4E), the mRNA cap-binding protein. This phosphorylation, performed by either MNK1 or MNK2 (MAPK interacting serine/threonine kinases 1 or 2), is believed to enhance the translation of a subset of mRNAs, many of which are implicated in tumorigenesis [reviewed by 5]. Using genetic and pharmacological methods to suppress eIF4E phosphorylation (*P*-eIF4E) we found expression of the proto-oncogene c-MYC is maintained by this signaling downstream of KRAS. Importantly, additional targeting of protein synthesis was required to reduce c-MYC levels, which correlated with suppression of proliferation. Specifically, a combination of rapamycin to suppress protein synthesis, and the MNK inhibitor eFT508 to suppress *P*-eIF4E-dependent translation, produced a dramatic reduction in proliferation and a more than 10-fold extension of survival in mice bearing *Apc*-deficient *Kras*-mutant intestinal adenomas. Supplementing our pre-clinical results, we analyzed clinical CRC samples and identified a positive correlation between mTORC1 and MNK activity, with nearly half of patients having elevated signaling through both pathways. Furthermore, stratification found a subgroup of these patients that had a 3.5-year shorter cancer-specific survival compared to those with low signaling through either or both pathways. This group accounted for 20% of patients studied.

eFT508 (clinical name tomivosertib) is an exceptional, well-tolerated compound that is progressing through eight clinical trials against multiple solid tumors. Our work provides pre-clinical validation for the combination treatment of eFT508 with rapalogues in *KRAS*-mutant CRC. We found similar effects when combining eFT508 with other compounds that suppress protein synthesis, such as oxaliplatin and 5-fluorouracil. eFT508 is currently being trialed in combination with paclitaxel in advanced breast cancer (NCT04261218) with paclitaxel known to suppress protein synthesis in cultured cells.^6^ Interestingly, genetic or chemical inhibition of *P*-eIF4E reverses chemotherapy-induced neuropathic pain induced by paclitaxel treatment *in vivo*, ^7^ indicating that there may be additional benefits of clinical targeting of *P*-eIF4E.

Mechanistically, we showed that *c-MYC* mRNA was significantly enriched in *P*-eIF4E immunoprecipitations from mouse organoids and human cell lines, identifying c-MYC as an essential effector of *P*-eIF4E. This is supported by our observation that increasing c-MYC expression *in vivo* was sufficient to completely reverse the benefit of our targeting strategy. Another recent publication found that the association of *c-MYC* mRNA with polysomes, the translation hubs of the cell, was significantly reduced in the human colon carcinoma cell line HCT116, where one copy of eIF4E could not be phosphorylated.^8^ This correlated with reduced c-MYC protein levels and cell growth. HCT116 cells harbor a mutation in *KRAS* (G13D) and activated Wnt-signaling via mutation of β-catenin (CTNNB1), making them similar to the *Apc*-deficient (Wnt activated), *Kras*-mutant mouse models used in our publication. Thus, the synthesis of c-MYC is highly dependent on *P*-eIF4E in two systems modeling CRC. Furthermore, the translation of c-MYC is undoubtedly important in a range of other tumor settings, making this targeting strategy potentially applicable to other MYC-dependent malignancies.

There is growing interest in targeting protein synthesis to suppress tumors. Many first-line chemotherapies suppress translation, indicating that this strategy has, in fact, been used for decades.^9^ During the search for targeted therapies that rely on biological understanding, the regulation of translation will be particularly amenable to therapeutic intervention. Targeting *P*-eIF4E provides an example of this, but clinical trials are currently underway using compounds targeting other parts of the translation machinery, providing clinicians with the tools to target translation initiation in cancer.

The rate of protein synthesis is increased by cancer-associated mutations in colorectal cancer, such as APC (APC regulator of WNT signaling pathway) loss or KRAS (KRAS proto-oncogene) mutation. Increased protein synthesis is permissive for hyperproliferation, with lower levels unable to support increased proliferation. However, it is also possible for excessive protein synthesis to occur, resulting in diminished proliferation and apoptosis. Tumor cells, therefore, tightly regulate their translation rate to remain in the permissive region. Therapies targeting protein synthesis can either decrease or increase overall rates. Many first-line chemotherapies suppress ribosome synthesis, and in turn translation, contributing to their efficacy as anticancer agents. These include 5-fluorouracil (5-FU) and oxaliplatin. More recently, targeted therapies against specific kinases regulating the translation machinery or translation factors themselves have been shown to suppress protein synthesis in pre-clinical models and have some efficacy in the clinic. Our recent article demonstrated the ability of targeting eIF4E (eukaryotic initiation factor 4E) phosphorylation to restore sensitivity to agents suppressing translation in *KRAS* mutant colorectal cancers. Targeted therapies are also being developed to increase protein synthesis rates in tumors, thereby overloading proteotoxic stress response pathways and reducing tumor proliferation. This strategy may be particularly effective against tumors with multiple mutations, where the permissive rate of protein synthesis may nearly be exceeded.
